# Decline of childhood overweight and obesity in Italy from 2008 to 2016: results from 5 rounds of the population-based surveillance system

**DOI:** 10.1186/s12889-019-6946-3

**Published:** 2019-05-21

**Authors:** Laura Lauria, Angela Spinelli, Marta Buoncristiano, Paola Nardone

**Affiliations:** National Centre for Disease Prevention and Health Promotion, National Institute of Health, Viale Regina Elena 299, 00161 Rome, Italy

**Keywords:** Prevalence trends, Children, Overweight, Obesity, Socio-demographic characteristics, Italy

## Abstract

**Background:**

Given the effects of childhood obesity on future health, and the lack of information of its prevalence in Italy, a national surveillance system was implemented in 2007. It is OKkio alla SALUTE, part of the WHO/Europe Childhood Obesity Surveillance Initiative (COSI). This study reports the 2008–2016 trends by sex, area of residence and socio-demographic characteristics in the prevalence of overweight and obesity in primary school children (8–9 years).

**Methods:**

In each round of the surveillance held in 2008, 2010, 2012, 2014 and 2016, a nationally representative sample of about 45,000 children, was weighed and measured with standard equipment and methods by trained personnel. Children were classified as normal weight, overweight or obese using World Obesity Federation (WOF) (formerly the International Obesity Task Force (IOTF)) and WHO cut-offs. Children’s sex, area of residence and mothers’ education and citizenship, were obtained using self-reported questionnaires and were assessed using multivariate logistic regression models.

**Results:**

Between 2008 and 2016, the overall prevalence of obesity dropped from 12.0 to 9.3% (WOF-IOTF) and from 21.2 to 17.0% (WHO), while the overall prevalence of overweight (including obesity) from 35.2 to 30.6% (WOF-IOTF) and from 44.4 to 39.4% (WHO). Reduction in the prevalence of overweight and obesity was greater in boys (− 14.5%, p for trend< 0.001; and − 24.7%, *p* = 0.001) compared to girls (− 11.1%, *p* < 0.001; and − 19.2%, *p* = 0.034). Decreasing trends were observed in overweight prevalences within children resident in the center and in the south. Decreasing trends in obesity prevalences were observed among boys resident in the north and in the south, and among girls resident in the center. Decreasing trends were observed in overweight prevalences within socio–demographic characteristics, except among children with low educated and foreign mothers; and in obesity prevalences for children with medium educated mothers, and girls with Italian mothers.

**Conclusions:**

From 2008 to 2016 a decrease of childhood overweight and obesity was observed in Italy. However, as these prevalences are still among the highest in Europe, there is need to continue their monitoring and implement more interventions to promote healthy lifestyles. More effort should be focused on children belonging to low social classes.

## Background

Childhood overweight and obesity have increased dramatically during the past decades, both in developing and developed countries and this presents one of the most serious public health challenges of the 21st century [1–3]. Although in recent years, the scientific literature suggests that the overall prevalence of paediatric obesity in developed countries may have plateaued [4], the prevalence of severe obesity is increasing [5, 6].

Childhood obesity is often persistent and increases the likelihood of lifelong health problems [7, 8] including cardiovascular disease, diabetes, musculoskeletal disorders, dyslipidemia, non-alcoholic fatty liver disease (NAFLD), hypertension and psychological problems [9–13]. Children who are overweight by kindergarten have a 4-fold greater risk of being obese in adolescence and obesity rates increase as children grow older, in particular when they become adolescents [14, 15].

A variety of factors have been found to be associated with obesity including sex, age, socioeconomic status, racial/ethnic groups, and geographic regions [16]. Boys have been found to be at greater risk of being obese than girls [17], but sex differences may vary by race/ethnicity as shown in a study on severe obesity in which Hispanic boys and non-Hispanic black girls had the highest prevalence of severe obesity [18]. Immigrant children and those of ethnic minorities are at higher risk of childhood obesity than native-born children [19].

Overweight rates are higher for children growing up in families with low income than for children from high income families [20]. Social disparities in obesity persist and are growing in some countries [21]. Socio-economic variability in childhood obesity and related lifestyles have also been reported in Italy [22] where the prevalence is lower in the north and higher in southern regions where socio-economic conditions and other health indicators are worst [23].

Varying cultural norms in feeding practices are also associated with children’s weight [24, 25], though this can be confounded by education and economic status.

Since 2007, following the recommendations of the WHO European Ministerial Conference on Counteracting Obesity [26], the Italian Minister of Health has promoted and funded the National Nutritional Surveillance System called “OKkio alla SALUTE” [27, 28]. The objective of this is to obtain information on nutritional status by routine measurement of bodyweight, height and lifestyle behaviour among primary-school children. This surveillance is part of the Childhood Obesity Surveillance Initiative (COSI) of the WHO Regional Office for Europe, which enables comparisons to be made between countries within the European Region [29].

The aim of this study was to examine the trends in the prevalence of overweight and obesity among primary school children (aged 8–9 years) from 2008/9 to 2016 using the WHO [30, 31] and World Obesity Federation (formerly International Obesity Task Force) WOF-IOTF [32, 33] cut-off values. Having obtained similar results with the two reference systems, further analysis of sex, geographical area of residence, mother’s educational level and citizenship used the WOF-IOTF definitions.

## Methods

The first data collection of the Italian National Surveillance System “OKkio alla SALUTE”, coordinated by the National Institute of Health (Istituto Superiore di Sanità), was in 2008 [34]. After, there were another four rounds in 2010 [35], 2012 [36], 2014 [37, 38] and 2016 [39]. The target population was children in the third grade of all primary schools, who are almost all 8 or 9 years old. At each round, with the support and assistance of the Ministry of Education, Universities and Research, lists of public and private schools and classes with the number of children in each class were obtained from regional school authorities. A stratified cluster sample design was used, following World Health Organization cluster survey methodology [40], with classes as the sampling unit. All children of the selected classes were invited to participate. All 21 Italian regions took part with representative samples at regional level or, if specifically requested, at Local Health Units (LHU) level were taken. Sample size was estimated in such a way as to achieve a precision of 3% in the estimate of the body mass index (BMI) for a regional estimates and of 5% for a LHU estimates. The finite population correction was used because the sample size was large relative to the school children population size, in particular when LHU estimates were made. The sample size estimate was also adjusted by assuming a design effect of 2 at the first round of data collection to take into account of the complex cluster sampling design. The value of the design effect found in two preliminary pilot studies for the BMI was very close to the unity; but we assumed a value of 2 to take account of other variables measured with the questionnaire which have assumed values up to about 3. The design-effect in each of the subsequent data collection cycles was estimated for each area of the country based on the results of the previous round. The value of the design effect for the BMI is variable among LHU or regions but almost always its value is no higher than 2.

According to the COSI protocol, children were weighed and measured with standard equipment (New Seca 872TM scales and Seca 214 stadiometers. Seca GmbH & co. Hamburg, Germany) and standard methods by trained local health staff. Children were weighed and measured after removing heavy clothing, and a simple checklist was used to describe the type of clothing that the child was wearing (the average weight of each type of clothing was used to adjust the measured weight for the analysis) [41]. Body Mass Index (BMI) was calculated and, according to the sex-specific WOF-IOTF cut-offs [32, 33], children were classified as overweight or obese. To facilitate international comparisons, the prevalences of overweight and obesity were also estimated using the WHO cut-off points [30, 31].

Children and their parents also completed two brief questionnaires. In the questionnaire addressed to the parents, information on their education (in 2008/9 this information was collected only with reference to the respondent parent) and nationality (not collected in 2008/9) was asked. The information from the two questionnaires was then linked through a unique identification code.

The questionnaires were available in Italian and nine other languages. The protocol was approved by the Institutional Ethical Board of the National Institute of Health and included the use of opt-out consent; that is parents were asked to specifically refuse participation, but the lack of a returned form was taken to imply consent to their child’s participation. Prevalences of overweight (defined as including obesity if not otherwise specified) and of obesity were the main outcome variables. The explanatory variables used for the analysis were: sex (males, females), area of residence (north, center, south), mother’s education (low = less than high school, medium = high school, high = university degree or more), citizenship (Italian, foreign). Differences in the prevalence according to these socio-demographic characteristics were tested by the Pearson design-based χ [2]. Weighted prevalences of overweight and obesity, with their 95% confidence intervals stratified by sex, were calculated for all the socio-demographic variables. Changes in the prevalence of overweight and obesity across the period 2008/9–2016 have been assessed using multivariate logistic regression analyses, taking account of the complex sample survey design. For each outcome of interest, a multivariate logistic model included the information about round of data collection as a categorical covariate. However, to investigate the linear time trend in the prevalence of overweight and obesity, multivariate logistic regression models were estimated that included a linear term for time, as well as all the socio-demographic variables. The significance of the trend was assessed using an adjusted Wald F test with α = 0.05. Separate models were used to test linear trends in prevalences by each stratification variable. Weights to adjust for the sampling design, oversampling and non-response were calculated and used to infer the results from the sample to the population. Moreover, all analyses took account of the effect of the complex survey design - namely clustering and stratification - on the estimation of the standard errors which were used to calculate the 95% confidence intervals. Data were analyzed using Stata statistical software version 11.

## Results

**Fig. 1:**
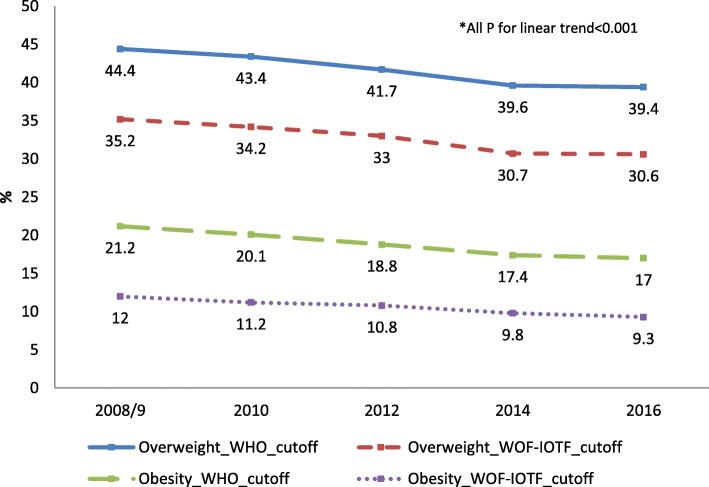
Time trends* in the Prevalence of Overweight and Obesity among aged 8–9 years children in Italy

The surveys involved about 45,000 children in each of the five rounds. About 3% of the parents refused authorization for the participation of their child. Sixty-six percent of the children were 8 years-old and 51% male. Forty-five percent were resident in the North of Italy, 22% in the Center and 33% in the South. Twenty-eight percent of the children had mothers with a low level of education, 48% medium level and 24% high educational level. Thirteen percent of mothers were foreign citizens. Figure 1 shows the time trends of WHO and WOF-IOTF estimates of the prevalence of overweight (including obesity) and obesity among 8–9 aged children. The different definitions of the cut-off mainly affect the prevalences of obesity, with WHO estimates 7–9 percentage points higher than WOF-IOTF estimates, almost the same difference is seen in the estimates of overweight. Between 2008/9 and 2016, the prevalence of overweight according to WHO, decreased from 44.4 to 39.4% (a relative change of 11.3%, p for trend< 0.001) and according to WOF-IOTF from 35.2 to 30.6% (− 13.1%, p for trend< 0.001). Obesity decreased from 21.2 to 17.0% (− 19.8%, p for trend< 0.001) and from 12.0 to 9.3% (− 22.5%, p for trend< 0.001) respectively. The WHO and WOF-IOTF prevalences of overweight not including obesity show a reduction from 23.2 to 22.4% (− 3.4%, p for trend = 0.01) and from 23.2 to 21.3% (− 8.2%, p for trend< 0.001) respectively. Subsequent analyses are based on WOF-IOTF estimates. Table 1 shows the time trends of the odds ratios for overweight and obesity controlling for the child’s age, sex and residence, and for mother’s education. The tests for the linear trend of overweight and of obesity are both statistically significant, *p* < 0.001.

**Table 1:** Multivariate analysis of overweight and obesity trends^a^

	*n*	Overweight			Obesity		
		OR	95%CI	*P* value	OR	95%CI	*P* value
Year of data collection							
2008/9	47,254	1			1		
2010	41,595	0.97	0.93–1.00	0.106	0.94	0.88–1.00	0.037
2012	45,421	0.96	0.92–1.00	0.037	0.96	0.90–1.02	0.185
2014	47,317	0.90	0.86–0.94	< 0.001	0.92	0.86–0.99	0.015
2016	44,193	0.90	0.86–0.94	< 0.001	0.88	0.82–0.94	< 0.001
Linear trend over the period 2008–2016							
	225,780	0.97	0.96–0.98	< 0.001	0.97	0.96–0.99	< 0.001

^a^Results represent logistic regression for complex survey data of the 5 rounds of “OKkio alla SALUTE” with reference to 2008/9 as well as results examining the time round as an ordinal variable controlling for children’s age and sex and for mother’s education and area of residence

The odds ratios by survey round show a similar but not always significant reduction of overweight and of obesity in 2010 and 2012 with respect to the reference round of 2008/9. The odds ratios are more marked in the last two rounds of data collection, in particular those regarding obesity. Moreover, the odds ratio and 95% confidence interval for overweight in the last round of 2016 are similar to the 2014 values, which may imply that a significant reduction in overweight occurred from 2008 to 2014 but little change occurred in the subsequent period.

### Overweight children

Table 2 shows prevalences of overweight by sex and socio-demographic characteristics. All prevalences show a decreasing trend between 2008 and 2016 except those related to children of foreign mothers.

**Table 2:** Prevalence of overweight (including obesity) among primary school children from 2008 to 2016 stratified by gender and maternal characteristics

	2008/9	2010	2012	2014	2016	Relative change 2016 vs 2008/9	*P* value for linear trend
	% (95% CI)	% (95% CI)	% (95% CI)	% (95% CI)	% (95% CI)	%	
Prevalence							
Boys	35.9 [35.1–36.8]	34.6 [33.8–35.5]	33.1 [32.3–33.9]	31.0 [30.2–31.8]	30.7 [29.8–31.6]	−14.5	< 0.001
Girls	34.3 [33.4–35.2]	33.7 [32.9–34.6]	32.4 [31.6–33.3]	30.5 [29.7–31.4]	30.5 [29.6–31.4]	− 11.1	< 0.001
Area of residence							
North							
Boys	27.6 [26.2–28.9]	27.1 [26.0–28.1]	26.7 [25.5–27.9]	24.9 [23.6–26.3]	25.1 [23.7–26.6]	− 9.1	0.085
Girls	25.7 [24.4–27.0]	26.9 [25.9–28.0]	26.2 [24.9–27.6]	24.5 [23.2–25.8]	25.4 [24.0–26.8]	−1.2	0.461
Center							
Boys	33.7 [31.9–35.4]	32.5 [31–34.1]	31.4 [29.6–33.2]	29.6 [28.1–31.2]	29.5 [27.7–31.4]	−12.5	0.001
Girls	33.6 [31.7–35.6]	31.4 [29.8–33.0]	29.5 [27.8–31.3]	29.3 [27.7–30.8]	29.9 [28.0–31.8]	−11.0	0.016
South							
Boys	44.2 [42.8–45.6]	42.8 [41.1–44.4]	42.5 [41.2–43.7]	41.1 [39.8–42.4]	39.5 [38.3–40.7]	−10.6	< 0.001
Girls	41.9 [40.4–43.4]	41.3 [39.7–42.8]	42.4 [41.2–43.7]	40.5 [39.1–41.9]	37.8 [36.5–39.1]	−9.8	0.002
Mother’s educational level							
Low							
Boys	39.2 [37.8–40.7]	37.3 [35.9–38.6]	37.9 [36.6–39.2]	35.9 [34.4–37.4]	36.7 [35.2–38.3]	− 6.4	0.212
Girls	38.4 [36.9–40.0]	36.7 [35.3–38.2]	36.9 [35.5–38.3]	35.3 [33.8–36.9]	36.1 [34.5–37.6]	− 6.0	0.286
Medium							
Boys	35.6 [34.2–37.0]	33.7 [32.5–34.9]	32.6 [31.5–33.7]	30.6 [29.4–31.8]	29.9 [28.7–31.2]	− 16.0	< 0.001
Girls	33.2 [31.7–34.6]	33 [31.9–34.2]	31.3 [30.1–32.5]	30.4 [29.2–31.6]	29.7 [28.4–30.9]	− 10.5	0.002
High							
Boys	30.3 [28.0–32.7]	30.0 [28.0–32.0]	25.0 [23.3–26.8]	24.8 [23.3–26.3	25.8 [24.3–27.4]	− 14.9	0.002
Girls	26.5 [24.5–28.6]	27.9 [26.1–29.8]	25.6 [23.9–27.4]	23.1 [21.5–24.7]	24.6 [22.9–26.4]	− 7.2	0.051
Mother’s citizenship^a^							
Italian							
Boy	–	34.9 [34.0–35.8]	33.4 [32.6–34.2]	30.9 [30.0–31.8]	30.2 [29.2–31.1]	− 13.5	< 0.001
Girls	–	34.3 [33.4–35.2]	32.6 [31.7–33.4]	30.9 [30.0–31.8]	30.5 [29.6–31.5]	− 11.1	0.002
Foreign							
Boys	–	31.1 [28.8–33.4]	31.0 [28.9–33.2]	31.5 [29.4–33.7]	34.4 [31.8–37.1]	+ 10.6	0.029
Girls	–	27.9 [25.8–30.2]	29.5 [27.1–32.1]	27.1 [25.1–29.3]	29.2 [27.0–31.4]	+ 4.7	0.825

^a^Information about mother’s citizenship was not gathered in 2008/9

The overall prevalence of overweight among boys across the 2008/9–2016 period shows a relative reduction of 14.5% (from 35.9 to 30.7%, *p* for trend = 0.001). Likewise, the overall prevalence of overweight among girls shows a relative reduction of 11.1% (from 34.3 to 30.5%, p for trend< 0.001). For boys, a statistically significant decreasing linear trend is observed for those resident in the center (*p* = 0.001) or in the south (*p* < 0.001), for those with a medium educated mothers (*p* < 0.001) or with a higher education (*p* = 0.002), and with Italian mothers (*p* < 0.001). For boys born to foreign mothers, the trend is statistically significant but increasing (*p* = 0.029).

For girls, a statistically significant decreasing linear trend is observed for those resident in the center (*p* = 0.016) and in the south (*p* = 0.002), for those whose mothers have a medium level of education (*p* = 0.002) and, although borderline, a higher level of education (*p* = 0.051), and were Italian (*p* < 0.001).

### Obese children

Table 3 shows prevalences of obesity by sex and socio-demographic characteristics. All trends are negative, except that of children born to foreign mothers for which no trend was detected.

**Table 3:** Prevalence of obesity among primary school children from 2008 to 2016 stratified by gender and maternal characteristics

	2008/9	2010	2012	2014	2016	Relative change 2016 vs 2008/9	*P* value for linear trend
	% (95% CI)	% (95% CI)	% (95% CI)	% (95% CI)	% (95% CI)	%	
Prevalence							
Boys	12.9 [12.3–13.5]	11.8 [11.2–12.3]	10.9 [10.5–11.4]	10.3 [9.76–10.8]	9.7 [9.2–10.2]	−24.7	0.001
Girls	10.9 [10.4–11.5]	10.5 [9.9–11.1]	10.2 [9.7–10.8]	9.4 [8.9–9.9]	8.8 [8.3–9.3]	− 19.2	0.034
Area of residence							
North							
Boys	7.6 [6.9–8.3]	7.9 [7.3–8.5]	7.0 [6.3–7.6]	7.1 [6.4–7.9]	6.0 [5.3–6.7]	− 21.4	0.042
Girls	6.5 [5.7–7.4]	6.8 [6.2–7.3]	6.3 [5.6–7.0]	6.2 [5.6–6.8]	5.9 [5.2–6.7]	− 9.5	0.672
Center							
Boys	10.8 [9.6–12.1]	9.6 [8.6–10.7]	8.8 [7.9–9. 9]	8.9 [8.0–9.9]	9.1 [8.1–10.3]	− 15.6	0.235
Girls	8.9 [7.8–10.2]	8.6 [7.6–9.7]	8.7 [7.5–9.9]	8.0 [7.0–9.1]	7.0 [6.2–8.0]	− 21.5	0.028
South							
Boys	18.5 [17.4–19.6]	16.7 [15.5–17.8]	17.4 [16.5–18.3]	16.0 [15.0–17.0]	15.5 [14.6–16.5]	− 16.2	0.017
Girls	15.7 [14.7–16.8]	15.0 [13.8–16.2]	16.4 [15.4–17.4]	15.1 [14.0–16.1]	13.8 [13.0–14.7]	− 12.1	0.172
Mother’s educational level							
Low							
Boys	16.7 [15.7–17.9]	14.6 [13.6–15.6]	14.6 [13.7–15.5]	14.9 [13.8–16]	13.8 [12.6–14.9]	− 17.4	0.141
Girls	14.4 [13.3–15.5]	13.3 [12.3–14.4]	14.1 [13.2–15.1]	13.4 [12.3–14.5]	12.6 [11.6–13.7]	− 12.5	0.443
Medium							
Boys	11.2 [10.3–12.0]	10.6 [9.8–11.5]	10.0 [9.3–10.7]	9.2 [8.4–9.9]	9.1 [8.4–9.8]	− 19.0	0.005
Girls	9.8 [8.9–10.7]	9.5 [8.8–10.4]	9.0 [8.3–9.8]	8.3 [7.7–9.0]	8.2 [7.5–8.9]	− 16.3	0.014
High							
Boys	8.1 [6.8–9.7]	6.7 [5.7–7.8]	5.6 [4.8–6.4]	5.4 [4.7–6.1]	6.2 [5.4–7.0]	− 24.4	0.096
Girls	5.0 [4.0–6.2]	5.8 [4.8–6.9]	5.4 [4.5–6.3]	5.1 [4.3–6.0]	4.8 [4.1–5.6]	− 3.6	0.912
Mother’s citizenship^a^							
Italian							
Boys	–	11.6 [11.0–12.2]	11.1 [10.5–11.6]	10.1 [9.5–10.6]	9.4 [8.9–10.0]	−18.7	0.061
Girls	–	10.8 [10.2–11.4]	10.3 [9.7–10.8]	9.4 [8.9–10.0]	8.9 [8.4–9.4]	−17.8	0.05
Foreign							
Boys	–	10.5 [9.0–12.1]	9.3 [8.0–10.7]	10.7 [9.2–12.4]	10.5 [9.1–12.2]	0.0	0.354
Girls		7.4 [6.1–8.8]	8.5 [7.1–10.2]	7.7 [6.6–9.1]	7.8 [6.5–9.2]	5.3	0.881

^a^Information about mother’s citizenship was not gathered in 2008/9

The overall prevalence of obesity among boys across the 2008/9–2016 period shows a relative reduction of 24.7% (from 12.9 to 9.7%, p for trend = 0.001). Likewise, the overall prevalence of obesity among girls across the considered period shows a relative reduction of 19.2% (from 10.9 to 8.8%, p for trend = 0.034). For boys, a statistically significant decreasing linear trend can also be observed for those resident in the north (*p* = 0.042) and in the south of the country (*p* = 0.017), and for those whose mothers have a medium level of education (*p* = 0.005). A borderline statistical significance was observed for boys whose mothers are Italian (*p* = 0.061).

For girls, there is a statistically significant decreasing linear trend for those resident in the center (*p* = 0.028), for those whose mothers have a medium level of education (*p* = 0.014) and Italian citizenship (*p* = 0.05).

The observed small reductions in prevalences of obesity, if interpreted as probabilities and transformed into corresponding absolute numbers of children throughout the Italian population aged 8–9 years, become very substantial. As an example, applying the WOF-IOTF prevalence of obesity in the 2008/9 (12%) and 2016 (9.3%) to the whole population aged 8–9 years resident in Italy in 2016 (*N* = 1,147,558), implies a reduction of about 31,000 obese children.

## Discussion

Our study provides evidence of a slight but persistent reduction of overweight and obesity in 8–9 year old children across the 2008/9–2016 period in Italy. The estimates are based on large nationally representative samples distributed by socio-economic characteristics similar to that observed in the general population of pupils attending Italian primary schools. The estimated reduction in the number of obese children aged 8–9 years in Italy is 31,000. If this percent reduction were the same in children of all ages (6–10 years) in Italian primary schools, the total reduction would be of the order of 15,000 children for each year of age. If the causes of the reduction were interventions of health promotion, information campaigns, etc., which are addressed to all primary school children (aged 6–10 years), this crude estimate may be plausible.

One objective of the monitoring of children’s weight is to increase the general awareness of the problem of overweight and obesity in primary school children and to promote interventions. In the past few years, several public events have been organized and media articles published to increase awareness of obesity and stress the importance of healthy lifestyle in Italy. Moreover, numerous interventions promoting healthy lifestyles, such as increasing physical activity and improving eating habits in the schools, have been implemented at regional and local level [36, 42].

The prevalences of overweight and of obesity among boys are higher than among girls across all the survey rounds, as is observed in most European countries [17], but the differences have diminished over time and are minimal in the 2016 round.

The stratified analyses of the prevalence of both overweight and obesity by socio-demographic characteristics show, from 2008/9 to 2016, reductions except for children of foreign mothers. Thus, children of foreign mothers, who had lower overweight and obesity prevalences in 2010, are comparable to children of Italian mothers in the last round 2016. This could be an indication of the cultural integration of the foreign children and families, which includes changes in their lifestyle and consequent higher risk of overweight and obesity.

The results regarding mother’s education deserve more consideration. The prevalences of overweight and of obesity are always higher for children of mothers with lower education and they do not show a statistically significant decreasing trend. The prevalences of obesity in children of low educated mothers are two-three times those of highly educated mothers. This implies persisting socioeconomic disparity in overweight and obesity and confirms the impact of parental education, as has been found by others [43].

Although in the last decade, policies and activities to reduce childhood obesity in Italy seem to have been effective, addressing the social determinants of health to move towards equity has only been marginally effective and important differences still exist. In part, this may be due to the fact that for socio-economically disadvantaged people, it may be more difficult to change unhealthy behaviours because their environment offers fewer opportunities and because it is more expensive to eat healthy foods [44, 45].

The prevalence of overweight and obesity is always higher in the south, the more economically disadvantaged part of the country, compared with the center and the north, although the reducing trends seem to slightly reduce these differentials. In our country, these geographic differences in the prevalence of overweight and obesity have also been found in the adolescent population [46]. For example, the WOF-IOTF prevalences of overweight (excluding obesity) among adolescents aged 11 years were: boys 14.5, 21.3, 28.5%; girls 11.6, 13.5, 21.1% in the north, center and south of the country respectively.

Both the overall high prevalence and the geographic disparities have profound implications for the country’s healthcare system now and in the future [47].

This study has some limitations. We have not considered important predictors of overweight and obesity in children, such as maternal BMI, which is correlated with children’s BMI and could be a confounder of the relationship between children BMI and the other socio-demographic factors considered in this study. We have performed many statistical tests without applying the Bonferroni correction. Results that are marginally statistically significant should be interpreted with caution.

The main strengths of this study are the representative population sample of school children throughout the country with more than 40,000 children and parents included in each survey and the use of standardized measurements of children’s weight and height. This large sample size allows stratified trends according to socio-economic characteristics to be investigated separately for males and females.

In this study, the overall prevalences of overweight and obesity based on the different WHO and WOF-IOTF cut-off criteria were calculated. Although some values, especially those related to obesity, show a big difference, with the WHO prevalence being 8–9 percentage points higher than WOF-IOTF prevalence, the trends show a similar pattern. This suggests, as has been also noted in other studies [48–53], that more attention should be paid to the definitions used when comparing overweight/obesity among various countries around the world, though the trend analysis tends to be independent of them.

Despite the observed reduction, the overall levels of overweight and obesity in Italy remain among the highest in Europe. In fact, within the COSI initiative, Italy has high prevalence rates of overweight and obesity with levels similar to those in Greece, Spain, Portugal and Slovenia [29].

## Conclusions

In Italy, from 2008 to 2016, prevalence rates of overweight and obesity among primary school children have significantly decreased. None-the-less, they are still among the highest in Europe. This implies that the number of overweight and obese children will continue to be a public health problem because quality of life and health in adulthood often depend on the habits adopted in early life. Interventions to reduce overweight and obesity and to promote healthy life styles need to be expanded and sustained and more effort should focus on children (and families) of lower social classes.

## Abbreviations

BMI: Body mass index
COSI: Childhood Obesity Surveillance Initiative
IOTF: Obesity Task Force
LHU: Local Health Unit
NAFLD: Non-alcoholic fatty liver disease
WHO: World Health Organization
WOF: World Obesity Federation
